# A goal-framing perspective on the important aspects of energy-efficient multifamily buildings

**DOI:** 10.3389/fpsyg.2022.926656

**Published:** 2022-07-26

**Authors:** Pimkamol Mattsson, Maria Johansson

**Affiliations:** Department of Architecture and Built Environment, Faculty of Engineering, Lund University, Lund, Sweden

**Keywords:** building end-user, design choice, dwelling choice, energy efficiency, goal-framing theory, multifamily building, professional

## Abstract

The growth of Sweden’s urban population necessitates new approaches for increasing the sustainability and energy efficiency of multifamily buildings. The development of such approaches will require a holistic and integrated understanding of the factors driving the decision making of both professionals who design buildings and end-users who live in them. This paper, therefore, uses the goal framing theory to determine which aspects of multifamily buildings are considered important by these two groups of actors. An empirical study based on semi-structured interviews with professionals involved in building design and development (project developers, housing company representatives, architects, and engineers; *N* = 15) was conducted to identify goals affecting the choices made during building design and development. In parallel, a questionnaire survey of building end-users (*N* = 61) was conducted to determine which factors guided their choice of dwelling. It was found that professionals’ design choices were primarily governed by normative goals relating to environmental benefits but were also influenced by the other goals. These included gain goals relating to budgetary constraints and keeping the building’s operational and maintenance costs low. Hedonic goals were also important; some design choices were made with the aim of providing pleasant, comfortable, and convenient living environments, or of giving the buildings a distinct aesthetic or some other special features. By comparing the professionals’ responses to the end-user surveys, it was found that the two groups had similar views concerning gain goals; both considered it important for apartments to be affordable and easy to maintain. However, their views on hedonic and normative goals differed markedly. The professionals sought to strike an optimal balance between different related aspects, whereas end-users placed greater importance on aspects relating to hedonic and gain goals when choosing dwellings. The findings provide a basis for constructive discussions on building design and development, and the scope for creating buildings that encourage end-users to adopt sustainable living practices while also satisfying their needs and preferences.

## Introduction

The growth of Sweden’s urban population means that there is a need for new ways of increasing energy efficiency in multifamily buildings, where space heating and domestic hot water account for a large proportion of the total energy use (Savvidou and Nykvist, 2020). Although efforts in this direction have been made by introducing new building regulations, new design strategies, and smart technologies, energy use in buildings has not declined significantly over the past two decades (the Swedish Energy Agency, 2019).

A combination of improvements in design and technology together with changes in the behavior of building end-users could substantially reduce energy use (Schweiker and Shukuya, 2010). It has been suggested that buildings could convey information about energy and behaviors through their design, form and energy-related features (Brown et al., 2009), and that green or sustainable building design could potentially foster pro-environmental attitudes and behaviors among users (Tucker and Izadpanahi, 2017). Moreover, the physical environment can have a “nudging” effect on energy-efficient behaviors, meaning that making changes in the physical environment can shape and encourage people within that environment to act in ways that increase energy efficiency (Lehner et al., 2016). Although it has become increasingly common to apply various energy-efficient solutions to both new and old multifamily building projects, previous studies have found that such solutions are typically considered optional (Storbjörk et al., 2018) or fail to challenge end-users’ energy-related behavior (Hagbert and Femenias, 2016). Failures to challenge end-user behavior were explained in terms of the fact that energy-efficient buildings were generally designed and built based on normative assumptions about housing standards and the comfort and convenience of end-users. A separate study showed that technical details rather than end-user behavior were the main focus of discussion among professionals involved in renovation processes (Palm and Reindl, 2016).

There is also the question of whether buildings with good energy performance can be considered sustainable if their users dislike them. For instance, an energy-efficient building envelope can substantially reduce energy consumption for heating but may also create problems in the indoor environment such as uncomfortably high indoor temperatures, leading to high cooling demands in summer (Tettey and Gustavsson, 2020). Energy-efficient technologies and appliances can even cause rebound effects that increase energy use in buildings (Alam et al., 2017). This may be due to user behavior changes, particularly if users believe that because the building is equipped with energy-efficient technologies or appliances, there is no need to think much about energy savings. A study on lighting controls (Maleetipwan-Mattsson, 2015) found that introducing automatic controls to switch-off lights can encourage habitual failure to manually switch lights off regardless of the availability of manual controls. The findings highlight the importance of integrated knowledge among building professionals and building users in the design and development of sustainable residential buildings (Janda, 2011; Hagbert and Femenias, 2016). It is also important to understand the expected performance of the buildings from both professionals’ and users’ perspectives in order to maximize energy savings.

Studies on professional perspectives (Zalejska-Jonsson et al., 2012; Hagbert and Femenias, 2016; Isaksson and Linderoth, 2018; Sandberg, 2018; Storbjörk et al., 2018) have highlighted several factors that are important in the design and implementation of energy-efficient multifamily buildings, including cost and financing considerations, norms relating to housing and material living standards, and knowledge about the benefits of addressing environmental considerations. However, it is still unclear whether the buildings can be expected to create opportunities for residents to adopt sustainable behaviors (Storbjörk et al., 2018). It was found that the most important issues for individuals looking to buy or rent an apartment were the apartment’s size, design, and location; energy and environmental factors had only a minor impact (Zalejska-Jonsson, 2013). A slight majority (56%) of individuals owning green apartments considered environmental certifications to be important and to therefore potentially influence a building’s attractiveness. A significantly lower proportion of owners of conventional apartments (39%) expressed similar sentiments. Among apartment renters, 40% of those renting green apartments considered energy and environmental factors to be important. There was no significant difference of opinion between individuals renting green apartments and those renting conventional apartments. The views of building end-users were broadly consistent with those of construction professionals relating to building norms.

Previous research on the perspectives of professionals and building end-users has focused on energy and environmental factors; other factors such as overall satisfaction and social environment have received less attention. More comparative studies are, therefore, needed to better understand the perspectives of these different actors and determine how well they are aligned. The results of such studies would provide a basis for constructive discussion about the energy efficiency of residential buildings from an integrated perspective and could thereby shape creative building design and development processes that support sustainable living and lifestyles. This paper presents an empirical study that was part of a research project investigating the design-building-user relation from the perspectives of professionals (building developers, architects, and engineers) and building end-users. Specifically, the paper aims to determine which aspects of modern energy-efficient multifamily buildings are considered important by professionals and building end-users. A theory-based approach rooted in goal framing theory (Lindenberg and Steg, 2007, 2013; Steg et al., 2014) is used to identify considerations that guide (i) professionals when making design choices about buildings and (ii) building end-users when choosing their dwellings, and to compare the views of these two groups. It was expected that the theoretical framework would clarify the important aspects of the buildings and make it possible to view the issue from an integrated perspective that accounts for the positions of both groups of actors.

### Goal framing theory

According to Lindenberg and Steg (2007), goal framing theory treats goals as the main determinants of how individuals perceive a given situation, process information, and act in response. Goals principally govern how individuals evaluate different aspects of a situation, what they focus on, and what alternatives are considered. Behavioral outcomes are usually influenced by multiple goals but may be primarily governed by just one.

Goal framing theory has been used to understand environmentally relevant behavior in specific situations including the adoption/rejection of energy-efficient solutions (Johansson et al., 2015; Gerdhardsson et al., 2018; Hameed and Khan, 2020). It posits that behavior is guided by three goal frames: (i) a hedonic goal frame associated with an individual’s desire to improve the way they feel (e.g., by seeking pleasure, excitement, or greater self-esteem) while avoiding negative thoughts or effort, (ii) a gain goal frame associated with the desire to gain (or avoid losing) resources such as time or money, and (iii) a normative goal frame associated with the desire to follow social norms (e.g., by contributing to energy reduction, combating climate change, or caring for others). These goal frames have been linked to a sustainable energy technology acceptance framework (Huijts et al., 2012) that proposes that acceptance/rejection of new technologies is based on the individuals’ evaluations of benefits, costs, risks and effects on the society or environment, and the resulting negative or positive feelings about the technologies. In any given situation, one of the three goal frames (the so-called “focal goal”) will have the strongest influence on an individual’s cognitive and motivational processes, and thus on their behavior (Lindenberg and Steg, 2013). Individuals make choices based on costs, risks or benefits when a gain goal is focal. If a normative goal is focal, decisions are based on effects on the environment or society, while feelings are the main determinant of decisions when a hedonic goal is focal. The degree to which a focal goal influences behavior is affected by other goals, its dominance is strengthened if it is compatible with other goals and reduced if there are conflicts with other goals (Steg et al., 2014).

A study on purchases of energy-saving air conditioners (Hameed and Khan, 2020) found that normative goals strongly influenced consumers’ intention to purchase the products, and that this effect was strengthened by hedonic goals. In this case, the influence of hedonic goals correlated with that of gain goals but had no significant effect on behavior. Hedonic goals of feeling good and normative goals of feeling obligated to act pro-environmentally were found to guide residents’ lighting choices at home, whereas gain goals of saving money were found to have little impact (Gerdhardsson et al., 2018). In a study on outdoor lighting choices, Johansson et al. (2015) found that a housing association’s decision to reject new energy-efficient outdoor lighting in a Swedish housing cooperative was driven by multiple goals. Among the association’s board members, the normative goal of perceived safety for elderly residents was weighted against the gain goals of avoiding costs and reducing energy consumption and therefore became the focal goal; the hedonic goal of improving perceived lighting quality seemed to have little influence. In contrast, the residents of the buildings were motivated primarily by the normative goal of improving perceived safety. Besides providing insight into the goals that governed the acceptance/rejection of the energy-efficient lighting, this study showed that goal framing theory could be used to compare the goal frames of actors who made the decision about the lighting technology (the board members) to those of its end-users (the residents).

To the authors’ knowledge, goal framing theory has not previously been applied to the different groups of actors whose choices influence the energy efficiency of multifamily buildings. Real-world studies adopting this approach could thus provide new insights from an integrated perspective and reveal features of energy-efficient multifamily buildings that promote their acceptance by both building design professionals and end-users.

### Objective

This paper’s objective is to provide insights into the goals that determine how different aspects of energy-efficient multifamily buildings are evaluated by professionals involved in building design and development and by the buildings’ end-users. Specifically, the paper seeks to identify the focal goals of both groups and to determine how they relate to other goals. The research questions addressed are:

- What goals influence professionals’ choices in building design and how are they related?
- What goals influence end-users’ dwelling choices and how are they related?
- To what extent are the goals important to building end-users aligned with those important to professionals?

## Materials and methods

The empirical study is based on semi-structured interviews with professionals involved in the design and development of residential buildings (including project developers, housing company representatives, architects, and engineers) and a questionnaire survey distributed to a group of building end-users (residents).

The interview questions (see “Procedure”) were designed to collect information on design choices which were considered and selected by professionals having different roles in the design and development of multifamily buildings, and drivers of the choices. Based on to Cockton’s (2013) work, a design choice was considered as a solution to a problem through features and qualities of the built environment and also, as a concept. These different forms of design choices were taken into account and addressed through different interview questions. Further, the interview questions were reviewed by a colleague with experience in the building industry to check whether the words used to express design choices are commonly used in the field.

The questionnaire survey (see “Procedure”) was used to collect information on how residents weight different aspects of their dwelling choices in relation to the goal frames. This approach was applied since it was not possible to conduct interviews with a relatively large number of residents within the framework of the project. The questionnaire items were developed based on the respective goals’ related aspects addressed by previous studies on the rejection/adoption of energy-efficient solutions (Johansson et al., 2015; Hameed and Khan, 2020). Two aspects were selected for each distinct goal frame to describe drivers of dwelling choice. Different aspects used in the previous studies on the roles of goal frames in the rejection/adoption of energy-efficient solutions were taken as a starting point.

### Interviews with professionals

Fourteen semi-structured interviews were conducted with 15 professionals involved in the design and development of multifamily buildings: 5 project developers or housing company representatives, 5 architects, and 5 energy engineers (two interviewees: an architect and engineer, participated in one interview together) who collectively worked for nine different companies including architectural, consultancy, construction, and municipal companies. Participants invited to participate in the interviews were identified as different actors playing diverse roles in the design and development of the buildings to achieve energy efficiency. Participants were recruited through the network at the University’s Department of Architecture and Built Environment and *via* referrals from the previous interviewees. They were initially contacted by sending an email invitation containing a document explaining the study’s background, aims, and procedure as well as the voluntary nature of participation, the applied data confidentiality procedures, and the study’s compliance with the EU’s general data protection regulations.

The interview participants (11 males and 4 females) had worked in the fields of housing development, building construction, or architecture in Sweden for at least 5 years, and some had worked in the field for more than 30 years. Their areas of expertise included housing design, sustainable building design, life cycle analysis, energy-efficient and environmental building design, building performance simulation, environmental certification systems, project management, building management and social sustainability issues.

#### Procedure

Interviews were conducted between March 2020 and September 2021. Before being interviewed, each participant gave informed consent to their participation and agreed that the interviews could be audio recorded. One participant refused to allow the interview to be recorded, so written notes were taken during the interview instead. The interviews were conducted online using video conferencing software.

Participants were first asked to summarize their background using questions such as *“Could you briefly tell me about your work?”* and “*How long have you been working in the field?.”* To capture design choices which have been applied to multifamily buildings, they were then asked to describe their views on the design and development of the buildings, with particular emphasis on the aspects and design principles that they consider most important. Typical questions asked during this phase of the interview were “*Could you describe your view on the development of energy-efficient housing in Sweden?,” “In your opinion, what are the most important aspects of today energy-efficient multifamily buildings?,”* and *“What do you regard as key design principles when working on energy-efficient multifamily buildings?”* To further explore specific design choices, the participants were next asked to describe the solutions they used to achieve energy efficiency using questions such as “*Could you tell me more about the solutions you have applied to achieve energy efficiency?”* Finally, they were asked about how they account for the role of building end-users during the processes, using questions like *“Do you consider the buildings’ occupants when working on energy-efficient multifamily buildings?”* and *“In what ways are the behaviors of building occupants accounted for when working on the buildings?”* The interviews lasted for between 40 min and 1 h.

#### Analysis

The recordings were transcribed by PM. The textual material was carefully read and analyzed using a deductive thematic approach (Braun and Clarke, 2006) to allow the mapping of goal framing theory onto the participants’ statements about the design and development of energy-efficient multifamily buildings and addressing the desires and behaviors of the buildings’ end users. In such processes, design choices were regarded as key concepts or design principles and solutions (including both design and technical solutions) that the interviewees had applied to the buildings.

The data were coded manually, and every statement pertaining to design choices made during building design and development was coded based on its alignment with the three core constructs of goal-framing theory, namely hedonic goals (related to feelings about the design choices in relation to user perceptions of living environments), gain goals (related to cost–benefit analysis and resource-and/or time-saving), and normative goals (relating to effects on the environment or society in the forms of environmental and social benefits). These goals have also been considered in relation to a sustainable energy technology framework (Huijts et al., 2012). If a statement aligned with multiple goals, it was coded under each one. To assess interrater agreement, selected statements from the transcripts were coded by the two authors in parallel and then compared to see if they were mapped to the same goals. This process indicated an initial interrater agreement of 93%, with 100% agreement after discussion between the two authors. The codes were then analyzed to identify patterns in the ways participants expressed their perspectives in relation to each goal frame and the relationships between the goal frames.

### Questionnaire survey among building end-users

#### Procedure

The survey was conducted between 2021 and the beginning of 2022; it was part of post-occupancy evaluation (POE) of four newly built energy-efficient or green-rated multifamily buildings. The main objective of POE was to investigate how building-end users experience and use the buildings in relation to energy use. The POE contained about 15 questions and 41 items. To keep the survey to a reasonable length, the number of items corresponding to different aspects of dwelling choices were limited. Questionnaires were sent out by mail to residents of the buildings together with a cover letter and return envelope. The residents’ names and addresses were retrieved from a public online database. A total of 319 residents received questionnaires and invitations to participate in the study (excluding undelivered mail) and 61 questionnaires were completed and returned, giving a response rate of 19.12%. The respondents included 27 males, 32 females, and 2 nonbinary individuals. Their ages ranged from 23 to 91 years, and their mean age was 53.48 years (SD = 17.83).

The residents’ evaluations of different aspects of their dwelling choice with respect to the goal frames were assessed using the question “*How important are the following issues when you chose your dwellings*?” The question was answered by assigning scores to the following six items, each of which corresponds to a distinct goal frame:

1. The building has a distinct style and character (a hedonic goal, i.e., seeking pleasure or interest).
2. The apartment has amenities that I appreciate (a hedonic goal, i.e., seeking pleasance, comfort, or convenience).
3. The apartment is affordable (a gain goal, i.e., minimizing a cost).
4. Maintaining the apartment is easy and requires little effort on my part (a gain goal, i.e., saving time and money).
5. The building is certified as an environmental building or equivalent (a normative goal, i.e., contributing to environmental sustainability).
6. The building has good common areas such as a laundry room and a bicycle room that can be used by all residents (a normative goal, i.e., relating to social benefits).

Participants rated the importance of these items on a five-point rating scale where 1 corresponds to “very unimportant” and 5 to “very important.”

#### Data analysis

Data were missing for item 2 (the apartment has amenities that I appreciate; missing data in 1.64% of responses) and item 3 (the apartment is affordable; missing data in 1.64% of responses). Since the data were not satisfactorily normally distributed (the *p* value obtained in the Shapiro–Wilk test was < 0.001) for all items, the non-parametric Kruskal–Wallis test and the Mann–Whitney U-test with the Bonferroni correction (giving a stricter alpha level of 0.05/6 = 0.008) were used to investigate the extent to which each goal drove the respondents’ choice of dwelling. In addition, a correlation analysis based on Spearman’s Rho (applying the exclude cases pairwise option) was performed to examine associations between the items associated with each goal. All statistical analyses were performed using IBM SPSS Statistics 28.

## Findings

Results obtained from the interviews with the professionals and the questionnaire survey of building end-users are first presented separately. Their results are then discussed jointly to determine whether and to what extent the perspectives of the professionals overlap with those of the end users.

### Goal frames guiding design choices

**Figure 1:**
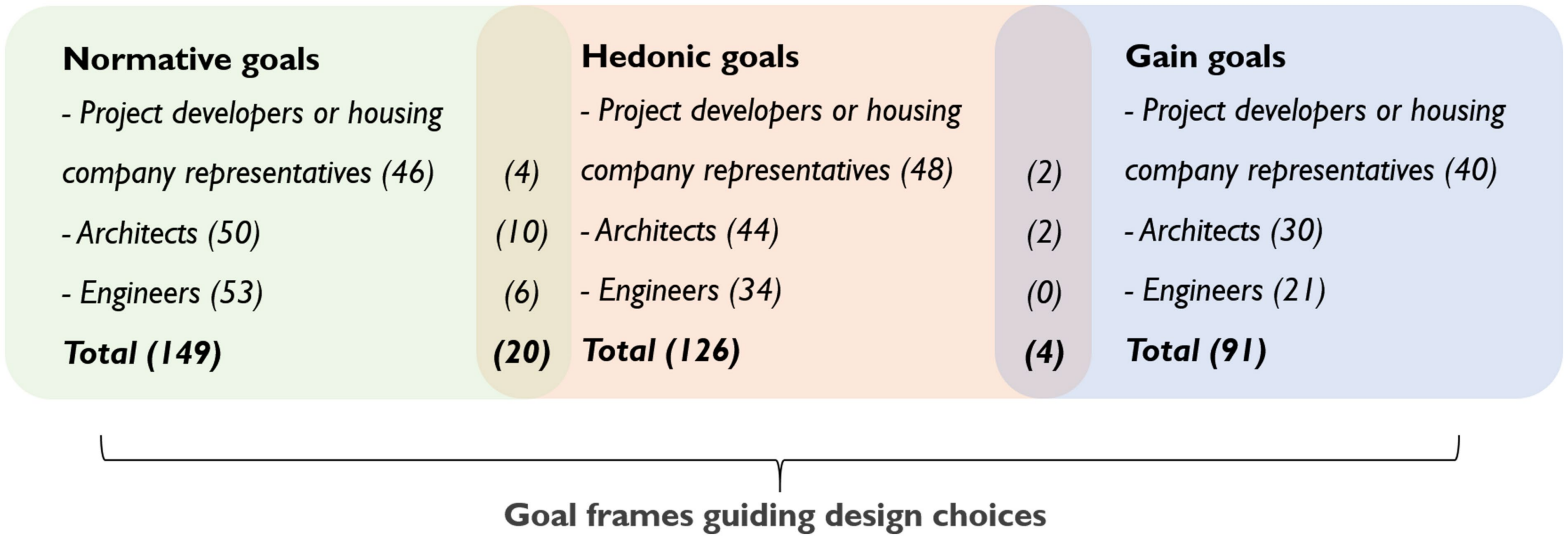
Numerical information indicates the frequency with which the participants expressed the goals. Note: overlaps were identified between hedonic and normative goals, and between hedonic and gain goals.

The results of the deductive thematic analysis are presented below. The frequency with which the participants’ statements pertaining to design choices tallied with each core construct of goal framing theory is presented in Figure 1.

#### Hedonic goal frame

The hedonic goal frame was based on feelings about design choices that were expressed during the interviews when discussing how the buildings’ end-users would perceive the living environments that the buildings offered. The issue most frequently mentioned as being important by professionals, particularly architects, and project developers or housing company representatives, was the perceived pleasantness of the dwellings, which was evaluated based on factors such as access to daylight, views, and noise levels. Other important hedonic aspects were the appearance and design features of the buildings’ exterior and interior, convenience, a good standard of living, and a comfortable indoor environment or climate. The latter factor was further characterized by a comfortable temperature, adequate ventilation humidity control, access to daylight, good acoustics, and a lack of harmful emissions from building materials. These aspects were frequently mentioned in relation to both common or shared areas and apartments.

*“The common laundry is on the first floor and have big windows…. We make the laundry room very inviting. It is nice and it feels pleasant to be in. If all machines are unoccupied, you can book up to six machines at the same time.”* (Housing company representative).

*“There are attempts to make apartments function well by considering how people use windows. I always try to provide space for a baby’s cot in the bedroom even it is ‘not a must’. I also try to create spaces that are controllable (by the building end-users) so people can sleep well and close the windows. (I) work to make sure people will feel good when they enter the apartment. I also try to make the kitchen feel as professional and usable as possible.”* (Architect).

These quotes show that positive feelings about design choices were associated with expectations concerning building end-users’ perceptions of the living environments’ pleasantness and convenience. Two participants (one architect, one engineer) also mentioned the need for privacy in this context, and took this need into account when making design choices.

*“We (architects) need to understand that ‘your dwelling’ is an automated place where you can be however you want to be. /…./. One person may want to have a lot of friends in their home and another may not want to host friends, and I think that being able to make that choice is a human right. So I could see kind of limit where you as an architect should not try to regulate behavior too much in the dwellings you create. So ideally, we (i.e., all actors) should perhaps think that ‘it is more about what we can share’ which is the most important thing.”* (Architect).

The quote above expressed a rather negative feeling about design choices intended to influence the behaviors of building-end users (and by extension, their privacy inside their apartments) while also placing greater focus on the buildings’ shared areas. However, some design choices were seen as having the potential to promote energy efficiency without affecting privacy or the availability of features enhancing comfort and convenience in apartments. For example, one project developer expressed positive feelings about a solution that aimed to reduce water use by changing behavior.

*“Individual measurement and charging means that tenants pay for the hot water or water that they use, and I think that’s a great tool. It means that residents learn the cost of hot and cold water, which is normally included in the rent. /…./. It will be clear to the users and could influence their behavior when they receive a bill or rent demand. For example, they might say “oh, I should not bathe every day because each bath consumes 4–5 liters of hot water and costs a lot of money – I should shower instead.””* (Project developer).

#### Gain goal frame

Economic considerations played a significant role in the selection of design choices particularly for project developers or housing company representatives and architects. Participants mentioned the need to avoid excessive design and investment costs. In general, using high quality materials and smart technologies or solutions to reduce energy use increases investment costs, which was frequently cited as a barrier to efforts to increase energy efficiency in multifamily buildings. The professionals preferred to avoid the extra costs of smart technologies or solutions in order to keep apartment prices affordable, and design choices were made with the aim of minimizing construction costs and financial risk. One housing company representative noted that in one case, a choice made when designing a building had been re-evaluated during the building’s use phase and was found to have been ineffective in the long term.

*“We measured and calculated that 30–35% of energy consumption was due to the central water system even though it was only used for a total of 10 min. Cold and warm water are so close, so I do not think the solution is good.”* (Housing company representative).

Four participants (two architects, one engineer, one project developer) stated that maximizing a buildings’ usability by ensuring that all of its functions are used has a positive impact on cost-effectiveness. If residents did not use the building’s functions in accordance with the intended design, the implication was that some of the money invested into the building had been spent ineffectively. Moreover, long-term cost effectiveness was considered when making design choices. In addition to energy costs, which were mentioned by most participants, there was a recognition that financial gains could be realized by reducing overall operational and maintenance costs during the buildings’ use phase.

*“For me it is all about costs - whether it is energy, money, time, or materials, it does not matter. All of them have to be considered together because if I waste a lot of energy, that is not sustainable. If I have a great product that is super energy efficient but breaks every year and forces me to buy a new one, that’s no good. So these things have to be considered.”* (Project developer).

This quote shows that the participant recognized that design choices can influence operating and maintenance costs that may not benefit only the buildings’ owners but the building-end users may also save time and costs for maintaining their own apartments.

#### Normative goal frame

In the context of this study, normative goals are goals pertaining to the environmental and social benefits of design choices. The chief environmental benefit was seen as reducing energy consumption and greenhouse gas emissions during the buildings’ use phase. All participants, regardless of their precise role in the design and development of the buildings, stated that their design choices relating to these issues were primarily made to comply with building energy codes and standards together with building regulations and guidelines. Five participants (one architect, one engineer, and three project developers or housing company representatives) also mentioned that organizational visions and goals pertaining to environmental sustainability played important roles and prompted efforts to design buildings such that they would receive green or environmental certifications.

*“Energy requirements always come from the National Board of Housing’s requirements…. but our requirements are based on the criteria for the ‘Environmental Building – Silver’ certification.” (Engineer).*

Participants believed that social benefits were realized through design choices that provide safe and secure living environments in accordance with building regulations while also promoting social inclusion and interaction between the building’s end-users. A couple of participants (two architects and one housing company representative) discussed efforts to promote social inclusion in a housing project by providing different forms of tenure and to encourage social interaction in particular through the common areas.

*“I have noticed that when we sit and talk about new housing projects that are going to be built, we talk a lot about common areas and reducing the size of the apartments to incorporate common areas where people can sit and work or do something and also meet and talk to their neighbors.”* (Housing company representative).

Additionally, most participants (five architects, two engineers, and four project developers or housing company representatives) saw their work on the design and development of multifamily buildings as a way of addressing sustainability challenges and the United Nations sustainable development goals. Some participants further suggested that the sustainability challenges facing society need to be taken into account when considering what makes a multifamily building sustainable. This has affected norms in the building industry and has therefore influenced design choices.

*“Over the last 10 years people have started talking about sustainability rather than energy saving, so the whole branch (of the firm) has begun looking at new practices, arguments, and requirements….”* (Housing company representative).

This quote shows that the design and development of multifamily buildings has moved beyond merely seeking improvements in energy use and is now focused on more holistic approaches for creating sustainable buildings. Participants also stressed the complexity of integrating the three dimensions of sustainability in practice.

In the participants’ statements (Figure 1), overlaps of the goals were identified, mostly between the normative goal relating to social benefits and the hedonic goals relating to convenient, pleasant or attractive living environments (12) in making design choices for shared or common areas. There were also overlaps between the normative goal relating to environmental benefits and the hedonic goals relating to the convenience, pleasantness or attractiveness (2) and the indoor comfort (1) of apartments, and the appearance as well as design features of buildings (1). The gain goals relating to cost effectiveness and to low operation and maintenance costs were found to overlap with the hedonic goals relating to comfort and convenient living environments (4). (Note: numerical information indicates the frequency with which the goals overlapped).

#### Relationships between goal frames and their influence on design choices

Overall, the interviews showed that the design choices made during the design and development of energy-efficient multifamily buildings were principally guided by normative goals relating to environmental benefits. This was reflected in statements about the need to comply with building energy codes and standards and to obtain green or environmental building certifications. Most efforts focused on reducing the energy used for space heating and domestic water heating. This was generally achieved by making design choices relating to the building’s orientation, form, ecological features, the materials used in the façade (including doors, windows, and insulation), interior walls, and systems for heating, ventilation, cooling, and providing hot water. Moreover, efforts were made to reduce environmental impacts by using solar panels to generate electricity for use in the buildings’ common areas and by installing energy-efficient lighting, equipment, appliances and fixtures. The behavior of building end-users was generally accounted for by performing energy calculations in which the expected number of users and their behaviors were quantified on the basis of reference values for variables such as occupancy hours, durations of window opening for airing, release of bodily heat, and consumption of domestic hot water and electricity. However, it was often noted that the values used in these calculations may not fully reflect reality. Normative goals relating to social benefits was also highlighted as drivers of design choices, particularly in relation to the buildings’ common or shared areas. Key objectives in this regard included ensuring easy access, pleasantness, and attractiveness to promote social interaction among the building’s end-users; such key objectives were associated with hedonic goals. Participants also emphasized the environmental benefits of the design of such areas (e.g., staircases, bike rooms, common laundry rooms, gardens, and trash rooms), which could potentially encourage energy-saving and environmentally friendly behaviors among building end-users.

**Figure 2:**
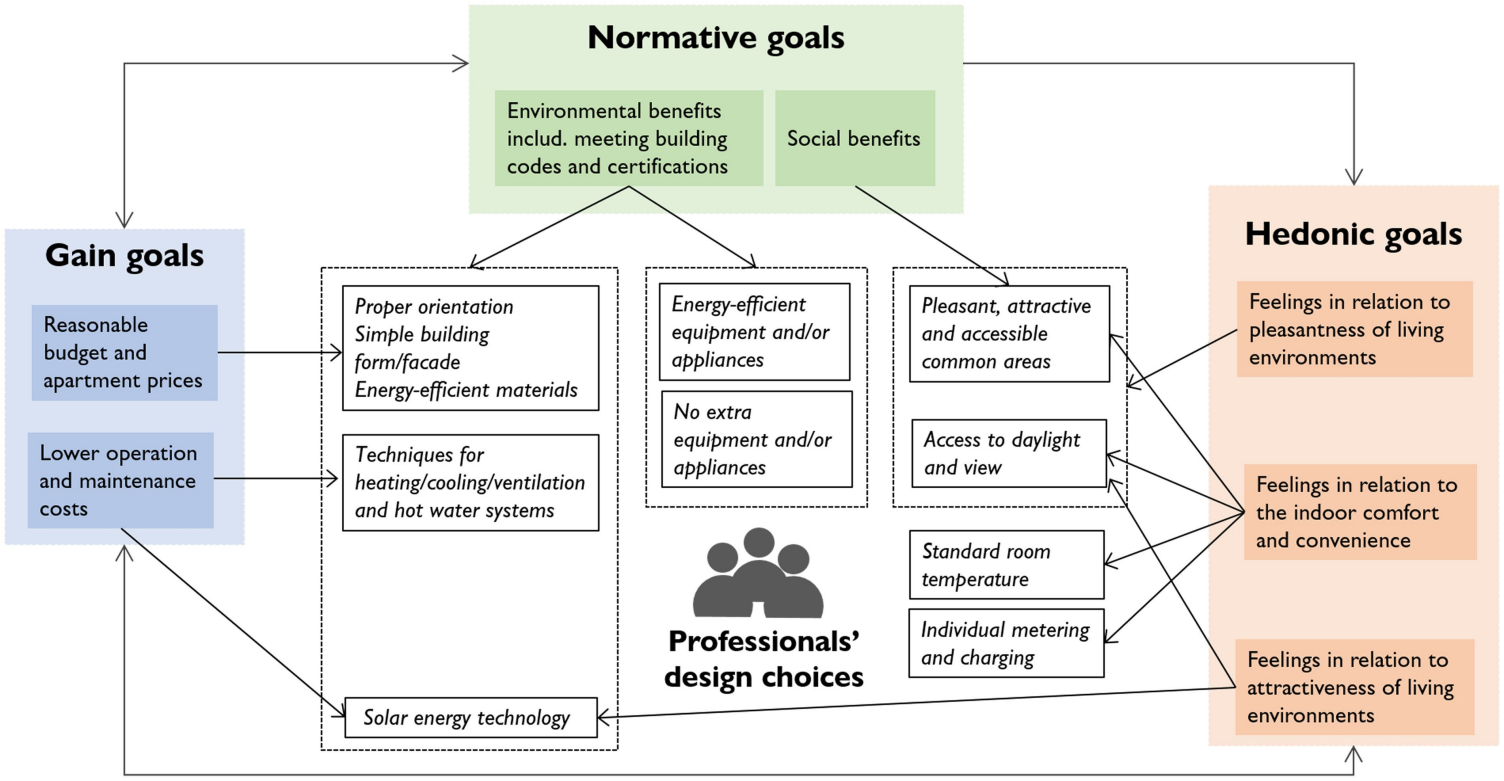
Relationships between the goals and their influence on design choices (indicated by the arrows between the boxes).

The relationships between the goals and their influences on design choices are illustrated in Figure 2. Although the normative goal relating to environmental benefits was found to play a dominant role when making design choices, it appeared that multiple goals were interrelated. Several participants stated that design choices were made with the aim of staying within a ‘reasonable’ budget to keep the apartments affordable (gain goals) while achieving acceptable levels of energy efficiency (normative goal) and providing good living environments (hedonic goals) for the building’s end-users. For example, one choice was made on economic grounds due to its long-term energy efficiency.

*“Insulating buildings well enough to reach the most demanding (energy) requirements is very expensive, but going 0 to 100 m^2^ of solar panels gives a much greater benefit.” (Engineer).*

This quote clearly illustrates how the participant weighted different design choices from an economic perspective, leading to a preference for solar panels. Most participants similarly expressed positive feelings about this choice regardless of their roles. Among other benefits, the inclusion of solar panels was seen as something that could trigger interest or excitement among prospective residents while also making them aware of the environmental benefits realized through the building’s capacity to generate its own energy. A couple of participants also described their awareness of the consequences of overproduction.

Further, the participants also felt that the normative goal relating to the environmental benefit sometimes created difficulties when trying to make design choices aligned with the hedonic goal of creating pleasant and attractive living environments.

*“In our projects that we have struggled to provide enough daylight, which is something that has become increasingly important. /…./ some municipalities now require daylight calculations for building permits. When dealing with very dense blocks where buildings are closely packed, the only way we can increase daylight is by increasing window areas. However, to reduce heating requirements, we have to reduce window areas, so there is a conflict that cannot really be resolved.”* (Engineer).

The quote above shows that the professionals were aware that the goal of minimizing energy demand for heating could impose limitations on the scope for maximizing the pleasantness of the living environment by providing good access to daylight. This was also seen as a new challenge relating to the normative goal frame because of the introduction of a requirement to perform daylight access calculations in order to obtain a building permit. The professionals also recognized that the need to comply with building energy codes (another normative goal) imposed constraints on the design and affected the buildings’ esthetics (associated with a hedonic goal), necessitating a simple building form with a minimal façade area.

*“There are lots of buildings with interesting forms in Europe and other places that we could not have here because of the need to comply with energy requirements, but I am so used to it (simple building forms) so I did not think about it.”* (Engineer).

Furthermore, some choices that could enhance the hedonic goals of comfort or convenience in the apartments had to be avoided:

*“We try to avoid using comfort floor heating in buildings because it causes very high energy consumption; it is almost impossible to include underfloor heating in low energy buildings.”* (Engineer).

*“Sometimes we do not even include dish washers - we tend not to put dish washers in rental apartments, those who rent install their own.”* (Architect).

Participants weighted the normative goal relating to social benefits differently when making design choices relating to shared or common areas. Some of them felt that such areas could be highly prioritized because of the need to satisfy challenging energy requirements.

*“The energy requirements are getting tougher, we really need to do a lot…. in one case we needed to reduce window areas by 25%, which was really hard because we wanted to have them (windows). We solved the problem by removing most of the windows from the staircase; those windows were large and looked nice, but we had to take most of them away.”* (Engineer).

As demonstrated in this quote, the pleasantness of a common area (a staircase) resulting from large windows providing ample natural light and a pleasing view was reduced to satisfy stringent energy requirements. Another participant mentioned that it was common to reduce the space allocated to common areas in order to provide a better standard of living inside the apartments. In contrast, the normative goal relating to social benefits was prioritized in rental apartment buildings, where living space was reduced to provide larger common areas that were intended to promote social interaction between the building’s end-users. Decisions about common areas were also sometimes guided by a gain goal frame.

*“…We talk a lot about it (common areas) – it used to be mostly about common areas for gardening but now it’s more about (the areas) where people can meet. /…./ We built the apartments in (neighborhood name). A three-room apartment is 59 m^2^, which is rather small, and we see that people may dislike that, but on the other hand it makes them (the apartments) cheaper. I am not sure, it might also reduce their energy consumption.”* (Housing company representative).

The normative goal relating to environmental benefits was also found to motivate design choices concerning common or shared areas that were intended to promote energy-saving or environmentally friendly behaviors through the perceived pleasantness, attractiveness, or accessibility of the areas.

*“We have stairs of course and they have natural light and are the simplest way to go up and down …. There are elevators too, but they are dark and boring so you only use them when you have to. The stairs are also visible from the outside and they are turned towards entrance balcony.”* (Architect).

This quote illustrates how the design choices chosen for common areas were expected to promote energy-saving behavior, i.e., using stairs instead of an elevator. Moreover, a design choice providing easy access to a bike room had been suggested as having a potential to facilitate biking, but had not been preferred considering gain goals.

*“People should have a bike room at the entrance so that they do not have to go down to the basement to get their bikes; they should feel that they can just get on their bike and go. These ‘soft’ things, they have not started coming in/…./. Putting a bike room above ground takes away space that could be used for apartments, which means losing a lot of money.”* (Architect).

In relation to a normative goal frame, participants also mentioned that there are attempts to reduce the environmental impact of buildings over their entire life-cycle, and that more holistic approaches to sustainable design and development of multifamily buildings are needed. Five participants (one architect, two engineers and two project developers or housing representatives) stressed that sustainable building materials and more efficient use of materials are becoming increasingly important issues when considering cost effectiveness together with energy efficiency and comfortable indoor environments during the buildings’ use phase. It is also increasingly recognized that the pros and cons of technological solutions must be evaluated holistically, and that it will become necessary to strike an optimal balance between these different considerations, which can be related to the three goal frames, by compromising on certain design choices.

*“I think these conflicts we have today may become more intense in future, so I think we will have to make compromises and say what is important here and why do we make certain choices, and if we have documented those discussion and compromises then we will be able to talk to the building regulators or city planning offices and say these are the problems, this is how we have solved them.”* (Architect).

### Goal frames guiding choices of dwelling

#### The importance of goal frames

Building end users assigned different importance scores to the six aspects of energy-efficient multifamily buildings that were described in the questionnaire (Table 1), and there were significant differences between the scores assigned to each item: *H*(5, *n* = 364) = 36.96, *p* < 0.001. The end-users assigned the highest importance score to the availability of apartment amenities (item 2), which is related to pleasantness, comfort, and convenience and is thus linked to the hedonic goal frame. The next most important aspects were easy and low-effort maintenance of the apartment (item 4) and the affordability of the apartment (item 3), both of which reflect gain goals. Less important issues were the availability of good common areas (item 6) and the green or environmental certifications of the building (item 5), which reflect normative goals. The least important aspect was the building having a unique style or character (item 1), which was associated with a hedonic goal, i.e., a feeling of interest or excitement.

**Table 1:** Mean and median importance scores, and mean rank, for the six items included in the questionnaire for building end-users.

Item	*n*	*M*	SD	Mdn	Mean rank
(2) The apartment has amenities that I appreciate (a hedonic goal)^*^	60	4.45	0.53	4.00	228.10
(4) Maintaining the apartment is easy and requires little effort on my part (a gain goal)^*^	61	4.26	0.75	4.00	207.52
(3) The apartment is affordable (a gain goal)^*^	60	4.15	0.80	4.00	193.33
(6) The building has good common areas such as a laundry room and a bicycle room that can be used by all residents (a normative goal)	61	4.02	0.85	4.00	176.80
(5) The building is certified as an environmental building or equivalent (a normative goal)	61	3.77	1.01	4.00	154.94
(1) The building has a distinct style and character (a hedonic goal)	61	3.67	0.83	4.00	135.23

* The score was significantly higher when compared to some of the other items.

The score for the hedonic goal of apartment amenities was significantly higher than that for the normative goals of good common areas: *U* = 1323.50, *z* = −2.86, *p* < 0.005, *r* = 0.26 and the building’s environmental certification: *U* = 1097.00, *z* = −4.13, *p* < 0.001, *r* = 0.38, and the hedonic goal of the building’s style and character: *U* = 833.50, *z* = −5.28, *p* < 0.001, *r* = 0.48. The score for the gain goal of easy and low-effort apartment maintenance was significantly higher than that for the normative goal of the building’s environmental certification: *U* = 1322.50, *z* = −2.97, *p* < 0.005, *r* = 0.27, and for the hedonic goal of the building’s style and character: *U* = 1108.50, *z* = −4.13, *p* < 0.001, *r* = 0.37. Moreover, the score for the gain goal of affordable apartment cost was significantly higher than that for the hedonic goal of the building’s style and character: *U* = 1249.50, *z* = −3.20, *p* < 0.005, *r* = 0.29.

#### Relationships between the goal frames relating to dwelling choice

The relationships between the aspects corresponding to gain, hedonic and normative goal frames based on the six different items on the questionnaire are presented in Table 2. There were few relationships between different items and goal frames, i.e., a moderate correlation between the two items corresponding to gain goals: *r_s_* = 0.48, *n* = 60, *p* < 0.01, and weak correlations between the normative goal of the building’s environmental certification and the hedonic goal of the building’s style and character: *r_s_* = 0.28, *n* = 61, *p* < 0.05, and the gain goal of affordable apartment cost: *r_s_* = 0.35, *n* = 60, *p* < 0.01.

**Table 2:** Relationships between the six building aspects and the three goal frames.

	Hedonic goal frames		Gain goal frames		Normative goal frames	
	(1) Building’s style and character	(2) Apartment amenities	(3) Apartment cost	(4) Time and cost of maintenance	(5) Green/environmental certification	(6) Common areas
(1)	1	0.25	−0.90	0.009	0.28^*^	0.13
		*n* = 60	*n* = 60	*n* = 61	*n* = 61	*n* = 61
(2)		1	0.009	0.34	0.21	−0.06
			*n* = 59	*n* = 60	*n* = 60	*n* = 60
(3)			1	0.48^**^	0.35^**^	0.45
				*n* = 60	*n* = 60	*n* = 60
(4)				1	0.11	−0.03
					*n* = 61	*n* = 61
(5)					1	0.16
						*n* = 61
(6)						1

* *p* < 0.05, and;

** *p* < 0.01.

### Comparing goal frames expressed by professionals and building end-users

The analysis of the professionals’ views revealed that the selection of design choices was driven primarily by a normative goal relating to environmental benefits, specifically, the need to comply with building energy codes or obtain environmental building certifications. Gain goals relating to controlling investment costs, minimizing operating and maintenance costs, and offering apartments at affordable prices were found to play significant ancillary roles in determining the energy efficiency of the designed building and the provision of good and attractive living environments insofar as possible. The professionals were aware that the choices they made to limit energy use could reduce the aesthetic quality, pleasantness, comfort, and convenience of the buildings’ living environments. The normative goal relating to social benefits was found to be particularly important in guiding design choices relating to the buildings’ common or shared areas, which were often made with the aim of promoting social interaction. The normative goal relating to environmental benefits also motivated design decisions to make common or shared areas accessible, pleasant, and attractive in order to facilitate energy-saving or environmentally friendly behaviors. The idea that buildings’ common or shared environments can have environmental benefits is supported by an earlier study on household energy use (Gram-Hanssen, 2013), which suggested that energy efficiency increases when more people share a living space. However, the professionals weighted the importance of the buildings’ common areas differently to the living environment inside the buildings’ apartments.

Whereas the building’s environmental certifications were the most important consideration for the professionals, they were among the least important factor governing the dwelling choices of the buildings’ end-users, who instead prioritized amenities, easy maintenance, and affordability. The latter two aspects both relate to gain goals and were found to be associated with each other. Moreover, their importance in end-users’ decision-making aligns well with the emphasis that professionals place on minimizing operating and maintenance costs and ensuring that apartments are affordable when making design choices that may affect monthly fees (for user who purchase apartments) or rents (for rental apartments). The buildings’ common areas were substantially less important than the amenities within the apartments in the end-users’ decision making. To some degree this finding is consistent with the professionals’ view that common areas are of lower priority than apartments, although this view did not apply to rental apartments, where professionals preferred to reduce the size of apartments to increase the size of the common areas for enhancing social interaction. The least important aspect according to the end-users was the building’s distinct style and character; this aspect was assigned a significantly lower importance score than all other aspects except the environmental and social aspects (which both correspond to normative goals). This outcome is consistent with the professionals’ belief that a building’s appearance must sometimes be sacrificed in favor of more important design choices.

It is notable that end-users assigned very different importance scores to two aspects linked to hedonic goals, namely, (i) the provision of apartment amenities, and (ii) the style and character of the building; there was no relationship between the importance scores for these two aspects. Accordingly, the comfort and convenience of the living environments played a greater role in the professionals’ design choices than the style and character of the building (it was not possible to evaluate the relationship between such aspects in the case of the professionals). However, professionals felt that one design choice—adding solar panels to a building—gave it a special character as well as the ability to generate its own energy, so this design choice was driven by a combination of normative, hedonic and gain goals. This design choice was seen as something with the potential to trigger interest or excitement among prospective apartment buyers or renters, and to communicate information about the building’s energy consumption and environmental friendliness. This may partly reflect the end-users’ perception that a building’s environmental certification was related to its style and character as well as the cost of its apartments, although these associations were rather weak.

Taken together, the general findings indicate a consistency between the views of professionals and building-end users on a gain goal frame relating to costs for living in the apartments, and that their views on normative and hedonic goal frames were rather inconsistent. The findings from the interviews suggest that the professionals strived to find optimal balances between the related aspects of normative and hedonic goals when working with the design and development processes of the buildings and the selection of design choices. On the other hand, the findings of the building end-users’ views suggest that the building end-users would pay more attention to apartment amenities which contribute to pleasantness, comfort and convenience of the living environments rather than common areas, environmental certifications and special appearance of the buildings when making dwelling choice. These findings suggest that new approaches to building design and development are needed to satisfy the needs and preferences of end-users while simultaneously encouraging them to adopt sustainable lifestyles.

## General conclusion

The paper aimed to clarify which aspects of modern energy-efficient multifamily buildings are considered important by the professionals who design them and by the buildings’ end-users who live in them. Using goal-framing theory (Lindenberg and Steg, 2007, 2013; Steg et al., 2014) as a framework, aspects that guide professionals’ design choices and end-users’ dwelling choices were identified and compared. The findings provide insights into the goals that govern how both groups of actors, who interact with the buildings at different stages of their life-cycles, evaluate different aspects of such buildings.

The interviews confirmed the roles of goal frames and revealed which aspects of energy-efficient multifamily buildings are of interest to professionals. These aspects were subsequently coded and linked to specific goal frames. The constructs of goal-framing theory were readily apparent within the participants’ responses and made it possible to construct a rich description of the goals that drive the design decisions taken by members of this group, irrespective of their individual roles in the process of building design and development. While the dominating goal frame was a normative goal relating to energy codes and environmental building certifications, a range of other goals also influenced the professionals’ design choices. In particular, the goal of maximizing environmental benefits was constrained by the gain goals of staying within a predefined budget and ensuring low operating and maintenance costs. The combination of these normative and gain goals limited the scope for satisfying hedonic goals relating to the quality of the living environment. These hedonic goals were related to building aspects such as aesthetics, pleasantness, attractiveness, comfort, and convenience. Another aspect subordinated to the dominant normative and gain goals was social interaction, which was related to a normative goal. A major challenge for the professionals was striking an optimal balance between reducing a building’s energy use and providing pleasant, comfortable, and convenient living environments. The relative importance of different goal frames appeared to vary between areas of the building; in particular, normative goals relating to social benefits appeared to have a stronger influence on design choices concerning common or shared areas of buildings than on choices relating to other areas. Taken together, the findings suggest that the professionals’ design choices were guided by multiple goal frames rather than just one. This outcome is consistent with the results of an earlier study that used goal framing theory to describe the goal frames that motivated the adoption/rejection of energy-efficient lighting (Johansson et al., 2015).

End-users living in energy-efficient multifamily buildings assigned different priorities to the different aspects of the buildings. The most important aspect driving the dwelling choices of the end-users was linked to a hedonic goal (i.e., seeking pleasantness, comfort, or convenience). However, the least important aspect for end users was also associated with a hedonic goal (seeking pleasure or interest from the building’s appearance). End-users also assigned high importance to two aspects that corresponded to gain goals, namely easy and low-effort maintenance (which corresponds to the gain goal of saving time and money) and apartment affordability (which corresponds to the gain goal of preserving economic resources). End-users considered both of these aspects to be more important than social and environmental aspects corresponding to normative goals.

Some aspects might not easily be interpreted as a singular goal frame. As an example easy and low-effort apartment maintenance would save end-users’ time and money, and was therefore considered to reflect a gain goal. However, it could also be interpreted as a reflection of a hedonic goal (avoiding having to invest substantial effort into maintenance). Similarly, the aspect of convenience could save end-users’ time, so one could argue that it should be associated with a gain goal but in this work it was instead grouped with pleasantness and comfort and linked to a hedonic goal. Regarding the different interpretations, we have assigned these aspects to the likely focal goal frames according to the principle that the long term consequences would weight higher in the choice of an apartment. This was based on the housing functions such as status symbol, self-representation, security and privacy that affect the quality of life (Pagani and Binder, 2021). For example, ones may value convenience the dwelling offers as an attribute of comfortable, safe, pleasant or attractive living environments (hedonic goal) rather than saving a few minutes during the day (gain goal). However, this shows that individual building aspects can be linked to multiple overlapping goal frames. Interestingly, although a relationship between the two aspects corresponding to gain goals was found, there were no significant relationships between any of the aspects corresponding to hedonic or normative goals. This is somewhat consistent with the analysis of the professionals’ views, which indicated that there was no clear connection between the pleasantness, comfort or convenience of the apartments and the appearance of the living environments. These results show that different aspects reflecting the same goal frame are not always related. On the other hand, it should be noted that the professionals did think about the relationship between environmental and social benefits when making design decisions; the design of common areas was mentioned as having the potential to influence both environmental and social benefits.

It is interesting to compare the view of professionals and building end-users on different aspects of energy-efficient multifamily buildings through different goal frames. Their views on the economic aspects reflecting gain goals were closely aligned, but the same was not true for aspects reflecting normative and hedonic goals. Our findings indicate that end-users generally consider pleasantness, comfort and convenience aspects (which reflect hedonic goals) to be more important than aspects reflecting normative goals. This is important when considering the question of whether buildings with good energy performance can be considered sustainable if they are disliked by their users (Hay et al., 2018). The professionals highlighted the challenge of striking an optimal balance between different buildings aspects, which is exemplified by their struggles to simultaneously increase energy efficiency and the quality of the living environment. This can be understood in terms of the relationship between the different goal frames and their effects on design choices. Professionals believed that some design choices could convey information to end-users about energy and encourage the adoption of energy-saving or environmentally friendly behaviors. However, the potential of design choices to trigger undesired rebound effects must also be accounted for.

A few remarks on the methods should be made. First, the view of building end-users on different aspects of dwelling choices was captured only by few items as part of the POE. In further studies, it would be desirable to develop the POE instrument to include more items to cover other aspects of the goal frames as well as to systematically investigate relationships among these items (e.g., by means of a factor analysis). Second, the survey was used to collect data from the users. Though the view of the users could not be directly compared to that of the professionals, interviews with building end-users would be desirable to obtain a more nuanced understanding and to obtain a basis for further item development. In line with Zou et al. (2018), conducting qualitative study would improve understanding of the quantitative results. Patterns among the goals identified from interviews would also improve the comparison of goal frames between the groups of different actors.

Moreover, sustainable choices could be influenced by other drivers such as habits, attitudes, personal norms, knowledge together with the physical and social contexts (e.g., Klöckner and Blöbaum, 2010; van den Broek and Walker, 2019). To identify individual and contextual factors that play crucial roles in fostering or hindering personal choices, such drivers and their relationships with the goals frames should further be examined. The role of social contexts of different building phases should also be taken into consideration. In line with Johansson et al. (2021), this would provide an opportunity of holistic evaluation capturing the effects of social processes on the individual actors and the goal frames guiding their choices. There are different forms of social processes that could influence how the individual actors look at situations and evaluate goals (Gifford and Nilsson, 2014; Yang et al., 2021). In further research, it would be beneficial to integrate other psychological factors and possible drivers of sustainable choices to the goal-framing theory. It would also be desirable to examine the relations between social processes and the goal frames, and attempt to systematically identify both physical and social characteristics of living environments that contribute to sustainable values of buildings and promote sustainable lifestyles through a holistic approach.

The present work clarifies the relationships between different goals and the challenge of striking an optimal balance between them when making design choices, especially in cases where multiple goals create contradictory requirements. Methods for overcoming such challenges may be relevant in efforts to meet more general goals outside the context of building design such as the UN’s sustainable development goals. As such, they could play important roles in motivating decision making. Overall, this work shows that goal framing theory is a powerful framework for understanding and integrating the perspectives of different actors who interact with buildings in different phases of their life cycles. In this way, it provides a basis for constructive discussion about the design and development of energy-efficient multifamily buildings that motivate their residents to adopt or maintain sustainable lifestyles and at the same time promoting healthy living environments.

## Data availability statement

The data analyzed in this study is subject to the following licenses/restrictions: Ethical guidelines, i.e., the participants were informed that the data will be collected and used for the specific research project and the results will be published. The informed consent did not include data sharing issues. Requests to access these datasets should be directed to pimkamol.mattsson@abm.lth.se.

## Ethics statement

Ethical review and approval was not required for the study on human participants in accordance with the local legislation and institutional requirements. Written informed consent from the patients/participants or patients/participants legal guardian/next of kin was not required to participate in this study in accordance with the national legislation and the institutional requirements.

## Author contributions

PM and MJ contributed to conception and design of the study, and conducted data coding. PM collected and initially analyzed the data, and wrote the first draft of the manuscript. MJ contributed to revision. All authors contributed to the article and approved the submitted version.

## Funding

The work was funded by a grant from the Swedish Energy Agency, grant number 2019-020830, P49576-1.

## Conflict of interest

The authors declare that the research was conducted in the absence of any commercial or financial relationships that could be construed as a potential conflict of interest.

## Publisher’s note

All claims expressed in this article are solely those of the authors and do not necessarily represent those of their affiliated organizations, or those of the publisher, the editors and the reviewers. Any product that may be evaluated in this article, or claim that may be made by its manufacturer, is not guaranteed or endorsed by the publisher.
